# Giant pseudoaneurysm mimicking as soft tissue tumor: a case report

**DOI:** 10.1093/jscr/rjag036

**Published:** 2026-02-05

**Authors:** Muhammad Fadli P Pahlevi, Hashfi F Raz, Andri Andi, Muhammad H R Siregar

**Affiliations:** Division of Cardiothoracic and Vascular Surgery, Faculty of Medicine, Universitas Sumatera Utara – RSUP Haji Adam Malik, Bunga Lau 17 Street, Kemenangan Tani, Medan Tuntungan 20155 Medan, North Sumatera, Indonesia; Division of Cardiothoracic and Vascular Surgery, Faculty of Medicine, Universitas Sumatera Utara – RSUP Haji Adam Malik, Bunga Lau 17 Street, Kemenangan Tani, Medan Tuntungan 20155 Medan, North Sumatera, Indonesia; Department of Orthopaedy and Traumatology, Faculty of Medicine, Universitas Sumatera Utara, Dr. Mansyur 5 Street, Padang Bulan, Medan Baru, 20155 Medan, North Sumatera, Indonesia; Department of Radiology, Faculty of Medicine, Universitas Sumatera Utara, Dr. Mansyur 5 Street, Padang Bulan, Medan Baru, 20155 Medan, North Sumatera, Indonesia

**Keywords:** pseudoaneurysm, soft tissue tumor, repair vascular surgery, hematoma

## Abstract

Pseudoaneurysms are false aneurysms that develop following arterial wall injury and are characterized by a contained hematoma with turbulent flow. While commonly presenting with typical vascular symptoms, in rare cases, they can mimic soft tissue tumors, especially in chronic post-traumatic settings. We report the case of a 23-year-old male who presented with progressive swelling in the right thigh. Initially suspected to be an aggressive soft tissue malignancy. Chronic pseudoaneurysms may present with features mimicking malignant soft tissue tumors both clinically and radiologically, especially in the absence of classic signs like pulsatility or bruit. magnetic resonance imaging findings, particularly flow artifacts and absence of solid enhancement, and bedside Doppler ultrasound are key diagnostic tools. Misdiagnosis can lead to inappropriate and potentially dangerous interventions.

## Introduction

An arterial pseudoaneurysm can occur at any arterial site after any disruption of arterial wall integrity, resulting in a locally contained turbulent blood flow forming a hematoma with a neck that typically does not close spontaneously beyond a specific size [1]. Common causes of pseudoaneurysms around the knee include blunt trauma, penetrating trauma, fractures, previous surgical procedures, infections, and in rare cases osteochondromas [2]. Clinically, it may present with pulsatile hematoma, pain, ecchymosis, or active extravasation. In chronic scenarios, once a fibrous capsule has been formed, it may present with a persistent flow communicating with the arterial lumen [3]. The clinical scenario may mimic a soft tissue tumor. In the lower limb, pseudoaneurysms mimicking a soft tissue tumor have been described arising from the popliteal artery, distal femoral artery, and the anterior tibial artery [4]. We describe a case of post traumatic pseudoaneurysm involving the deep femoral artery which was initially misdiagnosed as an aggressive soft tissue tumor.

## Case report

**Figure 1:**
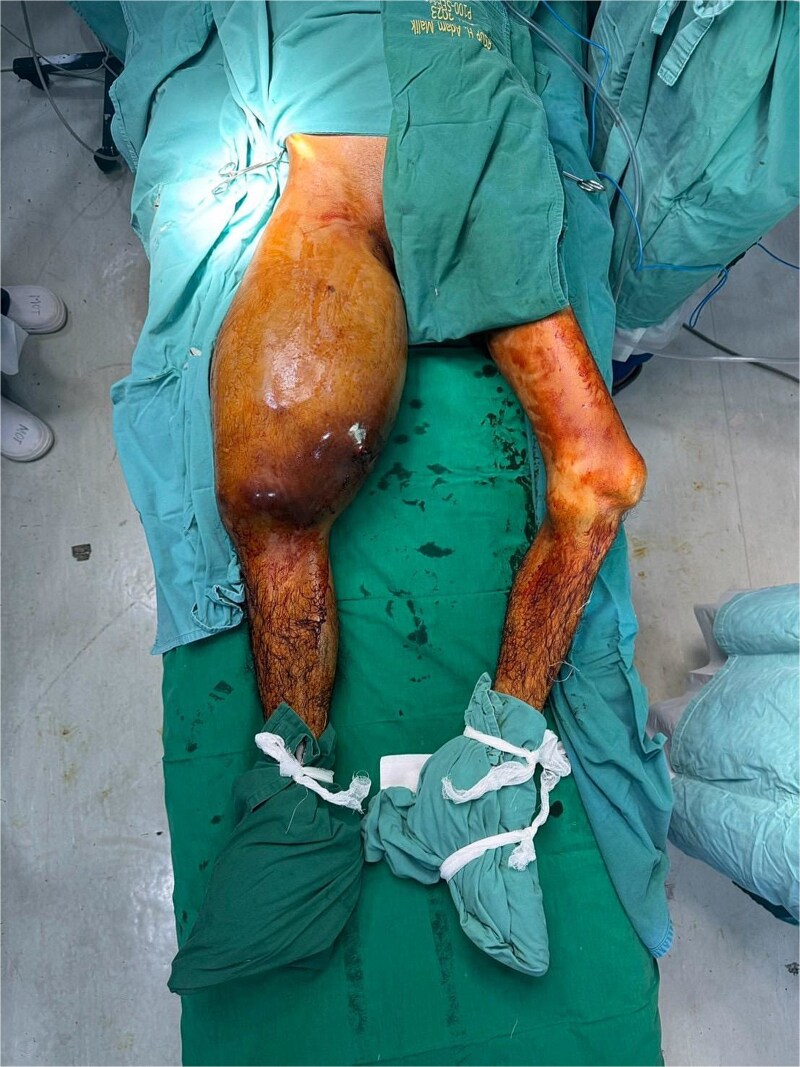
Clinical presentation.

A 23-year-old man presented with a complaint of swelling in the right thigh that had appeared one month prior. The mass was progressively enlarging without significant pain. The complaint was accompanied with discoloration of the overlying skin, the development of an ulcer, and bloody discharge. The patient also reported limited movement of the right knee, especially during flexion. There was no significant medical history, except for a blunt trauma about seven months earlier when he fell from a coconut tree. He only sought alternative treatment at the time, which led to temporary improvement (Fig. 1).

**Figure 2:**
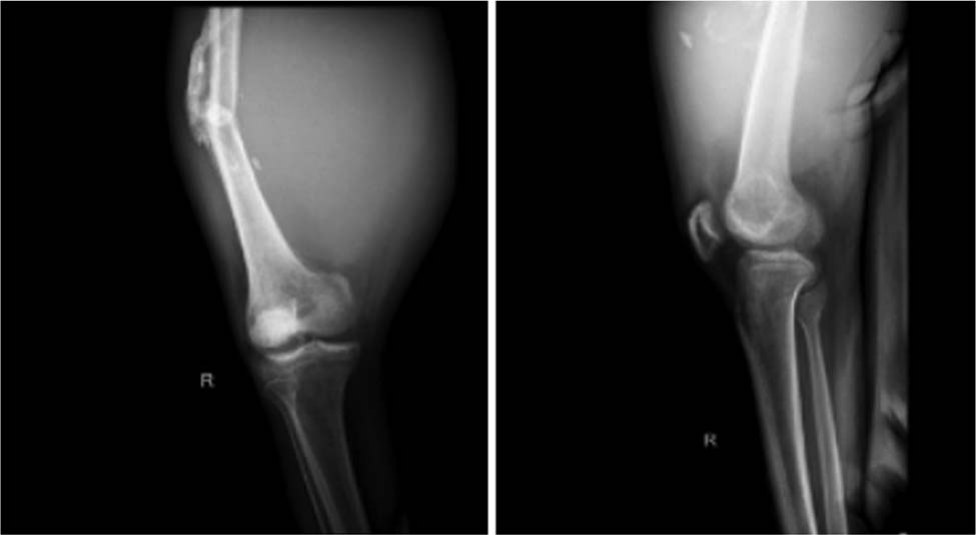
X-ray imaging of the right femur reveals a mid-shaft fracture with prominent callus formation. A soft tissue mass is observed surrounding the right femur, with no evidence of underlying bone lesions.

**Figure 3:**
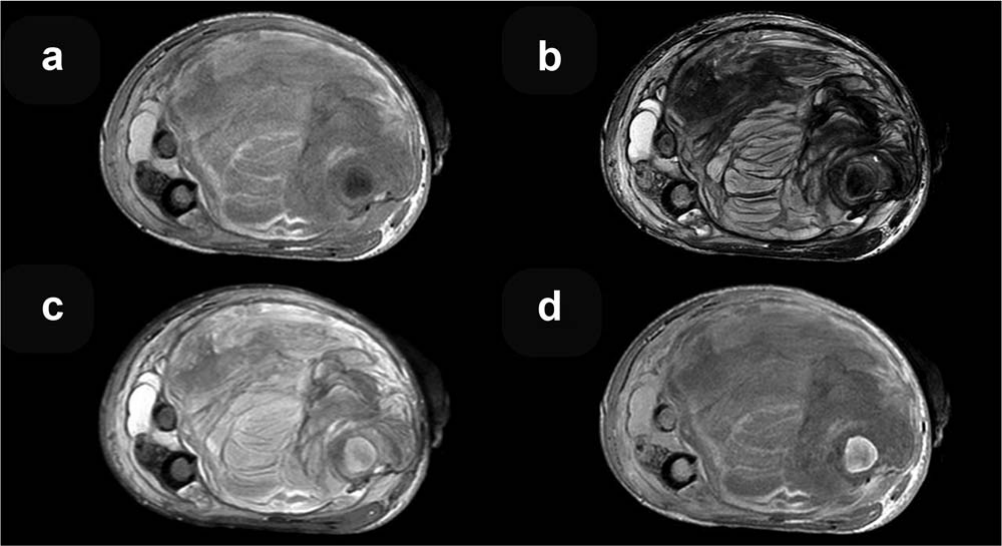
MRI axial sequences of the femur at the level of the fracture reveal a heterogeneous soft tissue mass. This mass exhibits predominantly iso-hyperintense signals on T1-weighted images (a), which, when correlated with T2-weighted images (b), are consistent with a hematoma of varying ages. The surrounding bone demonstrates normal signal intensity on T2 FS (c), and T1 contrast-enhanced (d) sequences.

**Figure 4:**
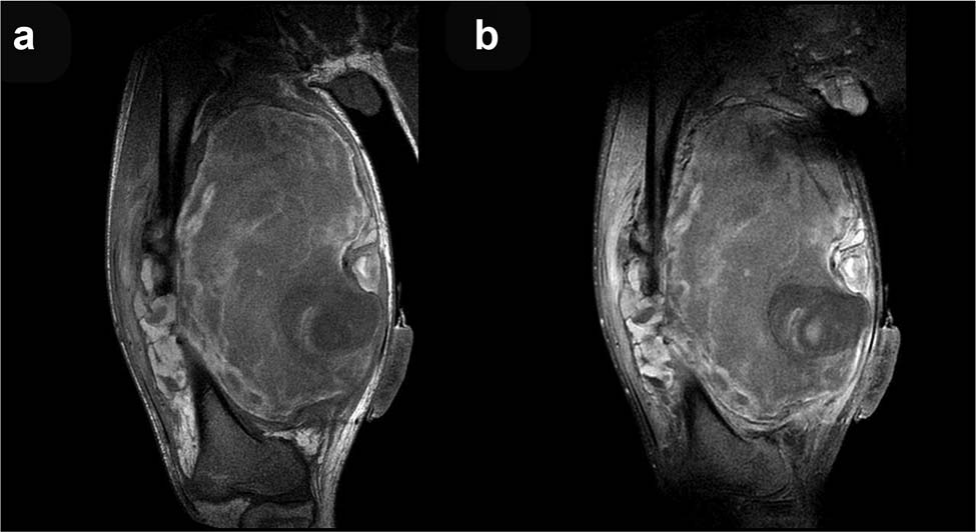
Coronal T1-weighted (a) and post-contrast T1-weighted (b) MRI sequences demonstrate no discernible contrast enhancement within the mass.

On physical examination, swelling was observed in the right thigh with a circumference of 56 cm, along with an open wound (ulcer) and bloody discharge. Palpation revealed a mass in the right thigh with a solid consistency, immobile, irregular surface, and well-defined borders. There was also a limitation in Active Range Of Motion (AROM) of the knee joint, especially during flexion. Pulsations of the femoral, popliteal, dorsalis pedis, and posterior tibial arteries were palpable. Radiology imaging are shown (Figs 2–4).

**Figure 5:**
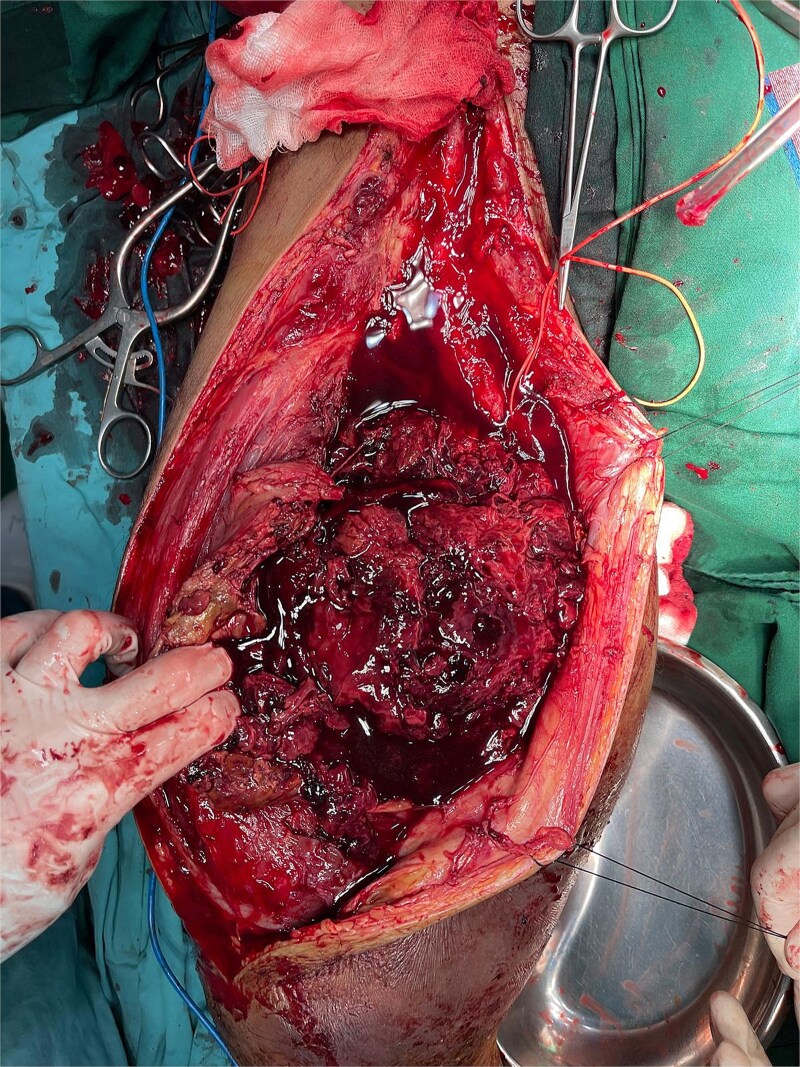
Intra operative finding.

**Figure 6:**
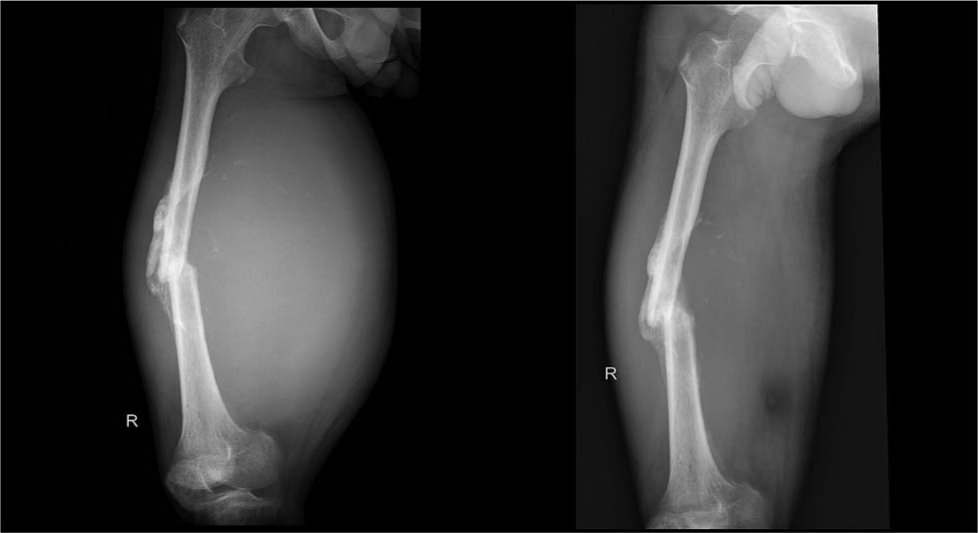
X-ray imaging post operation of the right femur reveals a mid-shaft fracture with prominent callus formation.

After administration of general anesthesia, standard aseptic, and antiseptic procedures were followed, posterolateral approach with vertical incision made on the right inguinal. Layer-by-layer dissection through skin, subcutaneous tissue, and fascia. Fibrotic tissue was identified and excised. Approximately 600 cc of pus, caseous tissue, and blood were drained. Lavage and sample collection performed. Femoral artery identified and controlled proximally. Incision extended and hematoma drained. A rupture was found in the profunda femoris artery, which was repaired using 6-0 Prolene. No leakage observed post-repair. Callus debridement and partial femoral bone resection were performed. Rectus femoris muscle was thinned, sartorius was ruptured, and vastus intermedius and adductors were poorly viable. Wound was sutured and a drain was placed. A tissue sample from the right thigh muscle shows only necrotic mass and hemorrhage in histopathology examination. There is an infiltration of inflammatory neutrophil cells among them. No signs of malignancy are observed. Patient was planned for more serial operations of debridement and Open Reduction and Internal Fixation (ORIF) because severe infection (Figs 5 and 6).

## Discussion

The diagnosis of a pseudoaneurysm is more straightforward in the setting of a pulsatile popliteal mass with bruit, and reduced ankle pulse. However, pulsation cannot be detected in some instance and a bruit is generally only associated with recent penetrating trauma. The region around the knee is the most common site for a post-traumatic pseudoaneurysm. Pseudoaneurysms are usually progressive and have complications, including thrombosis, embolization, and rupture [2].

This lesion gradually progresses in size due to constant arterial pressure and may mimic a soft tissue tumor or the reactive sclerosis with reactive periosteal reaction and associated soft tissue component which can in some time lead to suspicion of a bone tumor. The other common differential diagnosis of these lesions are hematoma, an arteriovenous fistula, lymphadenopathy, lymphocele, deep vein thrombosis, and compartment syndrome [5].

Knee is a common site for various soft tissue and bone swellings. These include neoplastic etiology like bone tumors, soft tissue sarcomas, pigmented villonodular synovitis, and nodal metastasis from lower extremity malignancies. Non-neoplastic pathologies that can cause similar swellings include infection, arterio-venous malformations, and pseudoaneurysms. It requires an experienced musculoskeletal radiologist to diagnose these lesions correctly. An incorrect diagnosis in these situations may be very deleterious to final outcome as it may cause major morbidity or mortality by inappropriate intervention [5].

The signs and symptoms of a pseudoaneurysm can mimic an osteogenic sarcoma. There have been case reports of popliteal artery pseudoaneurysm diagnosed as a soft tissue tumor and also a femoral artery pseudoaneurysm being diagnosed as malignant mesenchymal tumor.

Magnetic resonance imaging (MRI) can help in differentiating a sarcoma from a pseudoaneurysm. The pseudoaneurysm shows pulsation and flow artifacts, with T1 hyper intense areas due to associated thrombus and blood products. The dynamic contrast enhanced MR angiography shows the filling of contrast within the aneurysmal sac which confirms the diagnosis. Ultrasound with color tuler is a cheap, easily available bed side modality to diagnose pseudoaneurysm, though it is highly observer dependent.

This case is an example of a pseudoaneurysm mimicking a soft tissue sarcoma, as the patient a 23 year-old male presented with an initial complaint of progressive swelling in the right lower extremity, accompanied by ulceration, limited range of motion, and no significant pain. From radiological findings also which are features suggestive of a soft tissue sarcoma.
